# Association Between a History of Sexually Transmitted Diseases and Reproductive Health Knowledge Among Adolescents of Peru: A Cross-Sectional Study

**DOI:** 10.3390/ijerph23050613

**Published:** 2026-05-05

**Authors:** Jeel Moya-Salazar, Eliane A. Goicochea-Palomino, María Jesús S. Moya-Salazar, Magaly M. Medina-Rojas, Gloria Cruz-Gonzales

**Affiliations:** 1 GRINA, Faculty of Medicine, Universidad Señor de Sipán, Chiclayo 14002, Peru; majums2005@gmail.com (M.J.S.M.-S.); mmiriammr@uss.edu.pe (M.M.M.-R.); 2 Faculty of Health Science, Universidad Tecnológica del Perú, Lima 15007, Peru; elagoichi@gmail.com; 3 Faculty of Medical Technologist, Universidad Nacional Federico Villarreal, Lima 15007, Peru

**Keywords:** adolescents, sexually transmitted diseases, health knowledge, reproductive health, sexual behavior, contraceptives

## Abstract

**Highlights:**

**Public health relevance—How does this work relate to a public health issue?**

- The persistent incidence of sexually transmitted infections (STIs) and teenage pregnancies represents a substantial public health burden in low- and middle-income countries, affecting the holistic development of young people.
- Knowledge gaps and a lack of preventive information are critical factors that perpetuate adolescents’ vulnerability to reproductive health risks.

**Public health significance—Why is this work of significance to public health?**

- This study provides the first research in the Peruvian context to evaluate whether a prior negative health experience, such as an STI, influences knowledge acquisition and the adoption of protective behaviors in young people.
- The results reveal a critical and widespread confusion between the effectiveness of contraceptives in preventing pregnancy and their lack of ability to prevent infections, even in adolescents with a history of STIs.

**Public health implications—What are the key implications or messages for practitioners, policy makers and/or researchers in public health?**

- Sexual education programs need to be redesigned to explicitly differentiate between pregnancy prevention and infection prevention, using distinct teaching modules, terminology, and visual materials for each objective.
- Healthcare professionals must move beyond merely providing informational counseling and adopt interventions that transform clinical diagnoses into lasting behavioral changes.

**Abstract:**

Adolescents are prone to unwanted pregnancies and sexually transmitted infections. It is key that they receive reproductive sexual education during secondary education, which can be important for the prevention of these events. This study aims to compare knowledge of reproductive health between adolescents with and without STI history. A cross-sectional study was carried out on 164 schoolchildren from a national school in Lima (Peru). The AA-20 short questionnaire and the *t*-test were used to demonstrate differences between students with and without a history of sexually transmitted infections (STIs). Twenty-six (15.9%) students had STIs (mean age 16.6 ± 2.3 years). The average knowledge in students with and without STIs was 15.4 ± 3.7 points and 14.7 ± 3.9 points, respectively (*p* = 0.417). Among students with and without a previous STI, it was found that the majority used some form of contraception (61.5% vs. 31.9%, *p* = 0.004) and had received talks on the topic (92.3% vs. 72.5%, *p* = 0.031). Condom use was the most commonly used method to prevent STIs in both groups. In conclusion, students with a previous STI had slightly better knowledge about pregnancy and infections than students without a history.

## 1. Introduction

Adolescence is a critical developmental period characterized by profound physiological changes, including puberty and hormonal fluctuations, alongside complex social, academic, and vocational challenges [1]. This stage of transition also confers significant vulnerability to sexual and reproductive health (SRH) risks [2].

Globally, adolescents face a substantial burden related to SRH. The World Health Organization (WHO) estimates the adolescent birth rate at 4.9 births per 1000 adolescents, with approximately 11% of all births worldwide occurring in this age group. The majority of these births are in low- and middle-income countries (LMICs), where early marriage remains prevalent [1,3,4]. Concurrently, adolescents experience a disproportionate incidence of sexually transmitted infections (STIs), accounting for an estimated 26 million new cases globally in 2018 alone. *Chlamydia trachomatis* is a leading pathogen, causing about 1.6 million new infections annually in this population [5,6,7,8].

Despite targeted public health interventions [9], high rates of adolescent pregnancy and STIs persist, leading to adverse biological, psychological, and social sequalae. This persistent trend suggests that underlying factors beyond sexual behavior alone, such as gaps in comprehensive knowledge, inadequate education, and barriers to healthcare access, may be key contributors [10]. The absence of timely, organized preventive information places adolescents at significant risk for STIs and early pregnancy [11].

In Latin America, these challenges are critical, with persistently high rates of adolescent pregnancy and rising peaks of STIs, disproportionately affecting adolescents in LMIC settings like Peru [8]. The Peruvian context reflects this regional pattern, characterized by high adolescent fertility rates and significant STI incidence, underscoring a pressing public health concern [12,13]. While previous studies in Peru have assessed various determinants of STIs [12,13], a critical evidence gap remains. No study has specifically investigated whether a personal history of an STI or a significant reproductive health event is associated with differing levels of SRH knowledge among adolescents. Determining if such an association exists is crucial, as personal experience with a negative health outcome could theoretically influence knowledge acquisition and subsequent protective behaviors.

Therefore, we aimed to determine the level of reproductive and sexual health knowledge among school-attending adolescents in Lima, Peru, and to specifically compare knowledge levels between those with and without a history of STIs. The study’s main hypothesis was that high school students with a history of STIs have a higher level of knowledge about sexual and reproductive health.

## 2. Materials and Methods

### 2.1. Study Design, Population and Ethical Considerations

A cross-sectional observational study was conducted following the guidelines of the Checklist for Reporting of Survey Studies (CROSS) (Supplementary Materials Table S1) [14]. The study population consisted of all secondary education students from a public school in the district of San Juan de Lurigancho, Lima, Peru. This district is one of the most populous in metropolitan Lima, with over one million inhabitants, and is characterized by high poverty rates [15]. The Virgen del Carmen Educational Institution, the chosen school, runs an educational program that combines student growth and the development of critical thinking aimed at local social progress (based on socio-critical humanist theory) during secondary education [16].

This study was approved by the Institutional Ethics Committee of the Universidad Norbert Wiener (N-2020.03-01) and was conducted in accordance with the principles of the Declaration of Helsinki [17]. Written informed assent was obtained from all participating adolescents, and written informed consent was obtained from their parents or legal guardians.

### 2.2. Inclusion Criteria and Participant Selection

**Figure 1:**
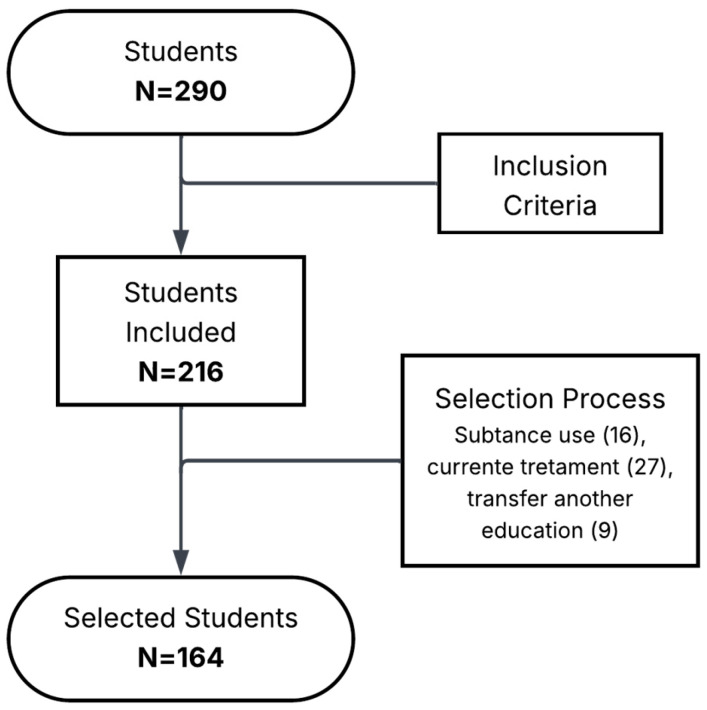
Participant selection flow diagram.

The inclusion criteria were adolescents of both sexes, currently enrolled in the school, who voluntarily agreed to participate in the study. From the total student population (*n* = 290), 216 students were initially eligible. Of these, 52 students were excluded due to recent transfer from another educational institution, substance use, or current treatment for mental health conditions (Figure 1). Students with substance abuse disorders and mental health problems received treatment from school staff and nearby health centers. That is why they were excluded with the goal of avoiding inclusion bias, since they could have had high knowledge in health and reproduction by being in medical care and counseling. We used a non-probability sampling method, which included the entire student population of the school. All students were included in a census to identify the final study sample.

### 2.3. Study Variables

The primary study variables were knowledge of reproductive health (including unintended pregnancy, types of contraceptives used, family planning, and safety of prevention methods) and sexual health (including history of STIs and participation in prevention workshops). The operational definition of a prior STI pertains to the clinical and diagnostic confirmation of any STI within the past two years, as reported by school principals during previous health campaigns. These variables were analyzed in relation to the students’ demographic characteristics.

### 2.4. Data Collection Instrument

The AA-20 questionnaire was used, a short, self-administered instrument developed by the non-governmental organization Nesh Hubbs in 2020. It comprises four closed-ended questions on reproductive and sexual health: (i) methods to avoid contracting STIs, (ii) safety of contraceptive methods, (iii) the number of sexual intercourses required for pregnancy, and (iv) consequences of adolescent pregnancy. The maximum score is 20 points, and a score ≥ 15 points indicates a higher level of knowledge about reproduction and infections.

The AA-20 consists of main questions, each worth 5 points, depending on the dichotomous response selected. The secondary questions aim to expand knowledge related to each dimension but are not scored. The score is calculated by summing the points for all four questions. The instrument’s developers established a score of 15 points as the threshold during its validation.

The instrument was culturally adapted for the adolescent population of Lima. The AA-20 questionnaire was validated in adolescent volunteers (average age 17 years) recruited by stakeholders. The AA-20 questionnaire was developed following standard validation guidelines, and an external reliability analysis was performed prior to its administration in this study [18]. It demonstrates excellent internal consistency (Cronbach’s α = 0.930), with all inter-item correlations being high and positive (range: 0.70 to 1.00), confirming its reliability in measuring the underlying construct (Supplementary Materials Table S2).

### 2.5. Data Collection Procedure

Data collection was carried out in July 2022 during the week of national anniversary activities, following coordination with the school administration. Prevention campaigns were held during the anniversary, so fieldwork was scheduled for that period. This does not mean there was an increase in students.

Students were invited to participate voluntarily in a face-to-face survey that complied with the institution’s established COVID-19 safety protocols. (An anonymous self-administered questionnaire was used, desks were spaced apart, classrooms were small, and teachers were absent). The average time to complete the questionnaire was 10 min. All collected forms were checked for completeness immediately after administration. The data were coded and entered into a Microsoft Excel 2013 spreadsheet.

### 2.6. Data Analysis

An initial descriptive analysis was performed, calculating frequencies for categorical variables and measures of central tendency for continuous variables (mean and standard deviation). The normality of data distribution was assessed using the Kolmogorov–Smirnov test. To compare reproductive and sexual health factors between groups, an independent samples *t*-test and χ^2^ was used. Correlations were determined using the Pearson test, with a *p*-value < 0.05 and a 95% confidence interval considered statistically significant for all tests. Then, we used multiple comparison correction with the Benjamini–Hochberg (BH) test. For effect measures, we used the Phi (φ) test and Cramer’s V as appropriate. Data analysis was performed using the SPSS statistical package version 24.0 (IBM, Armonk, NY, USA) and BoxPlotR (Tyers and Rappsilber Lab, Berlin, Germany) [19].

## 3. Results

### 3.1. Descriptive Data

A total of 164 students were included in the final analysis. The mean age was 15.9 ± 2.3 years, 66 (40.2%) were enrolled in the last year of high school, and over half were female (*n* = 92, 56.1%). The complete response rate was 100% of the students included in the study. Among the participants, 26 (15.9%) reported a previous STI, with a mean age of 16.6 ± 2,3 years, and 14 (53.8%) were male. Demographic characteristics of the participants are summarized in Table 1.

### 3.2. Knowledge Level

**Figure 2:**
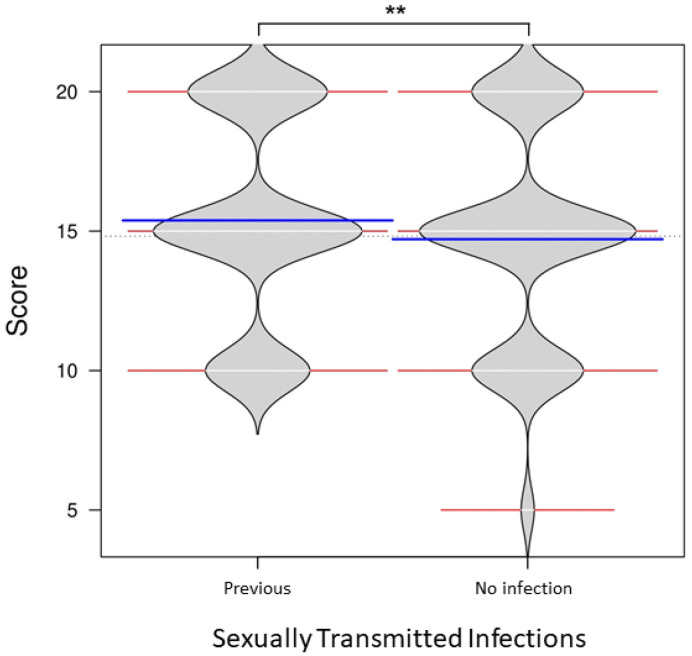
Knowledge scores on reproduction and sexuality among adolescents with and without a history of sexually transmitted infections (STIs). Median shown in line blue. ** *p* > 0.05 (non-significant).

The mean knowledge score was numerically higher in students with a history of STI (15.4 ± 3.7, 95% CI 14.0–16.8) compared to those without (14.7 ± 3.9, 95% CI 14.1–15.4); however, this difference was not statistically significant (*p* = 0.417). The distribution of knowledge scores on reproduction and sexuality is shown in Figure 2.

### 3.3. Behaviors

Analysis of reproductive and sexual behavior factors revealed several significant associations with STI history (Table 2). A significantly higher proportion of students with a previous STI correctly believed that pregnancy can occur after a single sexual intercourse (92.3% vs. 60.9%, *p* = 0.002). Furthermore, adolescents with an STI history were more likely to have used contraception (61.5% vs. 31.9%, *p* = 0.004) and to have attended educational workshops on the topic (92.3% vs. 72.5%, *p* = 0.031).

While not statistically significant, there was a trend suggesting that more students with an STI history perceived contraceptive methods as being 100% safe (61.5% vs. 43.5%, *p* = 0.091). Also, students with STI history were significantly less likely to consider abortion a solution (7.7% vs. 18.8%, respectively, *p* = 0.003).

The greatest differences between the STI and no STI groups were observed in the use of completely effective contraceptive methods (61.5% vs. 31.9%, respectively), abortion as a solution to teenage pregnancy (7.7% vs. 18.8%, respectively), the belief in becoming pregnant after single sex (92.3% vs. 60.9%), having received training on contraceptive and STI use (92.3% vs. 72.5%), and whether contraceptive methods are 100% effective in preventing STIs (61.5% vs. 43.5%). Furthermore, according to the multiple comparisons correction (BH), no significant differences were found between the groups that received lectures or training on this topic.

**Figure 3:**
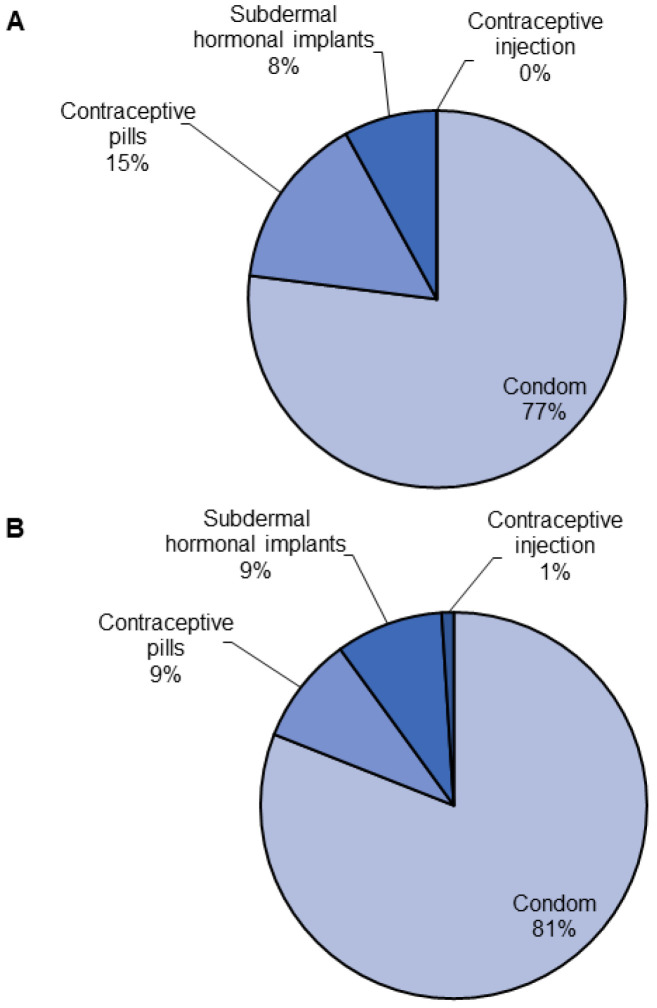
Frequency of prevention methods identified by secondary school students in Peru. (**A**). Students with STI history. (**B**). Students with no STI history. Data are presented as percentages.

Regarding STI prevention methods, the condom was the most frequently identified protective method by both groups (77% of those with an STI history vs. 81% without, *p* = 0.620). Contraceptive pills (15% vs. 9%) and subdermal hormonal implants (8% vs. 9%) were the second and third most frequently cited methods, respectively. There were no significant differences between the groups in their identification of prevention methods (*p* = 0.620). The frequency of all prevention methods chosen is summarized in Figure 3.

## 4. Discussion

This study found that adolescents with a history of STIs had a slightly greater understanding of reproductive factors and sexual behavior than those without previous infections. More notably, we identified significant differences in specific attitudes and behaviors as a history of STI was associated with a more accurate understanding of pregnancy risk from a single sexual act, a higher likelihood of contraceptive use, and greater exposure to formal sexual education. However, a concerning finding was the prevalent misconception, especially among those with an STI history, that contraceptive methods are 100% effective in preventing STIs, highlighting a critical confusion between pregnancy prevention and infection prophylaxis.

The overall moderate-to-high level of knowledge in our cohort is consistent with studies from Bosnia-Herzegovina [20] and Vietnam [21] where more than half had good knowledge but stands in contrast to reports of low knowledge from Ethiopia [22], Brunei [23], and the United States [24]. In Latin America, the results of this study are consistent with a study of students in São Paulo, which found that 87.7% knew what STIs were and 86.2% knew how to prevent them [25]. This disparity likely reflects vast differences in the implementation and quality of national sexual education programs.

There is evidence from around the world that teenagers with a history of STIs tend to have greater understanding scores. Studies in Tanzania [26] and Vietnam [21] found that personal experience with STI symptoms or prior education on the topic correlated with better knowledge. This aligns with the concept of “teachable moments,” where a salient health event motivates individuals to seek and retain information regarding treatment, transmission, and prevention [27]. This is supported by a German study of adolescents who underwent preventive youth health checkups, which reported better STI knowledge [27]. Also, having had one or two sexual partners improved STI knowledge in Ethiopian students [22], and highly educated adolescents were nearly 50% more likely to have good knowledge about STI transmission [24]. This factor suggests that both lived experience and formal education shape understanding [28].

In the other hand, our finding of a non-significant 0.7-point difference (15.4 vs. 14.7, *p* = 0.417) in overall knowledge scores is important despite its lack of statistical significance. It aligns with evidence that personal STI experience is an “inconsistent teacher”. Patients often seek information reactively during symptoms or diagnosis, but retention is poor without high-quality counseling [29,30,31]. Clinic-based education is frequently brief or unclear, leaving patients with persistent misconceptions even after treatment [32,33]. Consequently, personal experience may increase awareness of specific risks without substantially improving comprehensive, applied knowledge. This emphasizes that knowledge acquisition post-diagnosis is often partial and fails to bridge the gap to sustained behavior change [34,35,36]. The emotional impact of diagnosis may overshadow factual learning [30]. Therefore, our results reinforce that moving beyond informational counseling to skill-based, emotionally attuned education is critical for transforming personal experience into durable knowledge and safer practices [31,36].

A paramount finding of our study is the clear conflation of contraceptive and barrier methods. Many students, particularly those with an STI history, erroneously believed that hormonal contraceptives offer protection against infections, a misconception also reported in Ethiopia [22]. While condom recognition as a preventive method was high in our cohort, and other studies in Brazil [25] and Tanzania [24] with 91.8% and 97.2% of knowledge, respectively, this is undermined by evidence of its inconsistent use [37] and the failure of many adolescents to recognize its dual protective role [22]. This features a fundamental flaw in sexual health literacy; the distinction between pregnancy prevention and infection prevention is not being effectively communicated.

Other studies that are contradictory to our findings indicate low knowledge about STIs in 63% of Bruneian high school students [23] and similarly in German students with insufficient knowledge about STIs [27]. In American countries, it has also been reported that knowledge about STIs was low in late adolescents attending a university in the USA [22], and, in Peru, 82% of adolescents from the highlands answered incorrectly about the concept of STIs, demonstrating a low level of knowledge [38]. There may be differences between the populations evaluated, since there may be changes in the levels of education about sexuality and STIs between countries, and even within the same country.

Our finding that students with an STI history were significantly less likely to view abortion as a solution for young pregnant women (7.7% vs. 18.8%, *p* = 0.003) addresses a gap in the literature, as no identified study directly tests this specific attitude [39,40,41,42]. This novel association in a Peruvian adolescent population suggests that personal experience with one adverse reproductive health outcome (an STI) may shape perspectives on another (abortion). Potentially, the experience of diagnosis and treatment could foster a heightened valuation of pregnancy or an extra cautious attitude toward further medical interventions, though this requires confirmation. This finding is particularly salient in the Peruvian context, where adolescent pregnancy rates are high [43] and abortion is highly restricted [44]. It implies that adolescents with STI experience may constitute a distinct group with specific counseling needs; their reluctance to consider abortion may influence their decisions regarding contraceptive use or pregnancy resolution.

Within Peru, the literature reveals a trajectory of persistently low-to-moderate knowledge over the past three decades [4,12,13,45,46,47]. At the end of the 20th century, limited knowledge about sexuality was reported in students from four high schools in Lima [13]. A study of 1109 secondary school students at the end of 2007 found that 50% had little knowledge about STIs and sexuality [45]. Recently, several studies have found a low level of knowledge in secondary school students [12,38,46,47], although others have shown high and average levels of knowledge about pregnancy [4] and STIs [48]. Our findings contribute to this landscape by suggesting that a personal health event, such as an STI, may serve as a compound for knowledge acquisition [12].

Our findings must be interpreted within Peru’s context, where Catholic norms and restrictive sexuality education policies are prevalent. This atmosphere is associated with poorer SRH knowledge and persistent misconceptions among adolescents [21,49,50]. The observed confusion, where a history of STI was linked to both greater contraceptive use and the misconception that methods are 100% effective against STIs, likely reflects an educational framework focused on abstinence and pregnancy prevention while inadequately addressing infection prophylaxis [51,52,53]. Furthermore, the significantly lower acceptance of abortion as a solution among students with an STI history may be shaped by religious stigma limiting open discussion [49,54]. Overall, the pattern of specific knowledge gaps alongside reactive, experience-based learning aligns with evidence that restrictive, non-comprehensive sex education fails to provide accurate, retained, and applicable SRH knowledge, leaving adolescents vulnerable to misinformation [49,50].

Our results, therefore, reinforce the urgent need for enhanced, precision-based educational interventions [55]. Successful programs in Italy have demonstrated that targeted education can significantly improve knowledge of STI transmission, risk behaviors, and protective practices [56,57]. Similar educational interventions have shown promise in Peru [58]. An effective curriculum must explicitly dissociate pregnancy-focused methods from infection-preventing methods, leveraging the lived experiences of adolescents to consolidate knowledge and address persistent regional educational gaps [28].

### 4.1. Strengths

A key strength of this study is its novel focus on comparing knowledge levels between adolescents with and without a history of STIs, a dimension overlooked in prior Peruvian research [12,13,38]. The use of a validated instrument and a well-defined school-based cohort further strengthens the internal validity of our findings.

### 4.2. Limitations

This study has limitations. Its cross-sectional design precludes any causal inference regarding the relationship between STI history and knowledge acquisition. The sample was drawn from a single urban district and without a probability sample calculation, which may limit the generalizability of our findings, particularly to rural areas where knowledge and preventive behaviors may be substantially different [59]. The potential for social desirability bias in self-reported behaviors and knowledge must also be acknowledged. Most Peruvian studies have reported low levels of knowledge [4,38,47]; however, nationwide studies are needed to fully understand the level of knowledge among secondary school adolescent populations. Furthermore, there may be regional, generational, gender, and social differences among young people [60] in each country, so the results of this study may vary. Other limitations of the study include self-reported STI history and the potential unmodeled clustering effect (single school). Future research should employ nationwide, longitudinal studies to track knowledge and behavior over time and to better understand the causal pathways linking personal health experiences, education, and behavioral outcomes.

## 5. Conclusions and Future Directions

High school students with a history of STIs showed a slightly higher level of sexual and reproductive knowledge and were more likely to report preventative behaviors, such as contraceptive use and attendance at educational workshops, although these differences were not statistically significant. These findings require further research to support the results and explore other factors related to behavior and learning, both inside and outside of school. Despite the level of knowledge found, significant gaps persist, most critically the confusion between contraceptive efficacy and STI prevention. Sexual health education programs should explicitly differentiate between pregnancy prevention and infection prevention, using distinct visual materials, terminology, and teaching modules for each, rather than conflating all methods under general “protection”.

The critical importance of reaching adolescents with comprehensive education before they experience an STI could impact their reproductive health, given that our findings suggest only a modest knowledge advantage after STI occurrence. Future directions must include longitudinal studies tracking knowledge acquisition over time following STI diagnosis, qualitative research exploring how adolescents with STI history seek, process, and retain sexual health information, and intervention studies testing whether education that explicitly separates pregnancy from infection prevention improves both knowledge and behavior.

At the educational level, health institutions need to implement actionable curriculum-level initiatives, deepening the inclusion of disease prevention and control topics in science, technology, and health courses, particularly as to STIs, since students are immersed in the changes inherent to adolescence. In this sense, culturally adapted and curriculum-enhanced programs should be incorporated as a part of the educational plan in coordination with secondary education management units and the Ministry of Education. This will allow the integration of the health-prevention-education pillars that are so vital today.

## Figures and Tables

**Table 1:** Demographic Characteristics of the Study Participants (*n* = 164).

Variable	Category	*n*	%
Sex	Female	92	56.1
	Male	72	43.9
Age Group (years)	13–15	86	52.4
	16–18	68	41.5
	19–20	10	6.1
School Grade	Third	54	32.9
	Fourth	44	26.8
	Fifth	66	40.2

**Table 2:** Knowledge and Attitudes Regarding Reproduction and Sexuality Among Secondary School Students, Stratified by History of Sexually Transmitted Infections.

Item	STI History (*n* = 26)	No STI History (*n* = 138)	*p*-Value	*p* Adjusted	φ/V
Believes pregnancy can occur after a single sexual intercourse	24 (92.3)	84 (60.9)	0.002 **	0.016 **	0.246
Has ever used any contraceptive method	16 (61.5)	44 (31.9)	0.004 **	0.010 **	0.230
Has received talks or training on this topic	24 (92.3)	100 (72.5)	0.031 **	0.062	0.166
Considers contraceptive methods to be 100% safe	16 (61.5)	60 (43.5)	0.091	0.146	0.128
Considers abortion a solution for young pregnant women *	2 (7.7)	26 (18.8)	0.003 **	0.012 **	0.215
Has been spoken to at home about family planning	20 (76.9)	100 (72.5)	0.640	0.640	0.141
Is aware of the consequences of early pregnancy	26 (100)	132 (95.7)	0.282	0.376	0.132
Has a relative or acquaintance experiencing early pregnancy	8 (30.8)	50 (36.2)	0.596	0.681	0.150

Data in *n* (%), ** *p*-value < 0.05 (significant). *p* adjusted with Benjamini–Hochberg test. Abbreviation: Cramer’s V test: V. ** Response categories for this item were “Yes,” “No,” and “Maybe.” Maybe response: 6 (23.1%) and 62 (44.9%) for STI history and No STI history adolescents, respectively. The *p*-value is for the overall difference in response distribution.

## Data Availability

These research data are confidential under the relevant agreements and regulations. Public disclosure or sharing is not permitted. The data are securely stored at the NGO Nesh Hubbs. Authorization is required to access the data. For questions or information, contact Jeel Moya-Salazar at jeelmoya@gmail.com.

## Supplementary Materials

The following supporting information can be downloaded at: https://www.mdpi.com/article/10.3390/ijerph23050613/s1, Table S1: CROSS Checklist; Table S2: Case Processing Summary and Statistical Analysis.
